# Translation and Validation of the Greek Version of the Evidence-Based Practice Competency Questionnaire for Registered Nurses (EBP-COQ Prof©)

**DOI:** 10.3390/nursrep12040069

**Published:** 2022-09-27

**Authors:** Stefania Schetaki, Evridiki Patelarou, Konstantinos Giakoumidakis, Alexandra Trivli, Christos Kleisiaris, Athina Patelarou

**Affiliations:** 1 Department of Nursing, Faculty of Health Sciences, Hellenic Mediterranean University, 71410 Heraklion, Greece; 2 Intensive Care Unit, NIMTS Veterans’ Fund Military Hospital, 11521 Athens, Greece; 3 General Hospital of Agios Nikolaos, 72100 Lasithi, Greece

**Keywords:** evidence-based practice, nurses, competence, validity

## Abstract

(1) Background: Evidence-based nursing is the benchmark of the nursing profession. It is widely recognized that evidence enhances knowledge, skills, and competencies in nursing education and thus the quality of patient care. Although several proposals have been introduced to reinforce nurses through education in evidence-based practice (EBP) for clinical practice, there is no validated EBP competence tool to determine how nurses approach evidence-based nursing in healthcare practices in Greece. Therefore, the aim of the present study was to translate and validate the Greek version of the Evidence-Based Practice Competency Questionnaire, Professional version (EBP-COQ Prof©). (2) Methods: A cross-sectional study was conducted in a sample of registered nurses working in the public sector. (3) Results: 414 clinical nurses were recruited in total with a response rate of 75.3% and a mean age of 43 years old. Most of the nursing personnel were females, 354 (85.5%), and there were 60 (14.5%) males. Confirmatory factor analysis (CFA) showed a confirmation of the developer’s four-factor design. The estimated association between items on each scale showed a strong relationship. The competence questionnaire showed a high internal consistency between the components of attitude, knowledge, skills, and utilization. (4) Conclusions: The competence questionnaire shows a strong relationship between four the components, highlighting the four factors that should be promoted to improve the use of EBP nursing practices.

## 1. Introduction

Nurses face a complex health care status which explains the necessity for the development of up-to-date clinical practices in the current clinical era. It is commonly accepted that evidence-based practice is the critical element of integrated nursing care [1]. By searching for evidence and current practices, nurses can develop effective interventions based on updated and valid scientific knowledge [2]. Evidence-based practice, as a process, is the collection, interpretation, and integration of valid, clinically correct data. Globally, many efforts have been made to improve the provided patient care of healthcare systems [3,4]. Evidence-based nursing is the tool that is prioritized in this process [5]. Health stakeholders across the world have focused on the strength of the implementation of evidence-based nursing practices. The results show that the key element in linking evidence into practice is to act at both the local and the global level [6]. The vision of EBP should be shared with all nurses to strengthen healthcare systems.

In searching for the evidence of nursing competence for EBP, it has been determined that there is a lack of evidence in this field in Greece, and notably, there was no tool found in the Greek language for investigating the EBP competencies of the registered nurses. The international literature shows that in this scientific era, the Evidence-Based Practice Competency Questionnaire, Professional version (EBP-COQ Prof©) [7] is a valid psychometric tool which is designed to analyze the influence of different variables on the field of competency and is a sequence of research that many others have studied on the level of EBP readiness in various cultural domains [8,9].

The priority of this study is to validate a research tool in a large Greek sample of nurses, reflecting the need to fill an existing research gap [10,11] and utilize it in future research purposes as an accurate psychometric instrument. The validation of the EBP-COQ Prof Greek version was tested in the current study in a large nursing sample with all the appropriate statistical methods. Therefore, developing the proper assessment tools to evaluate EBP competence is a challenging process that includes adequate learning progress [12,13]

## 2. Materials and Methods

### 2.1. The Questionnaire

The Evidence-Based Practice Competency Questionnaire for Professional Registered Nurses, (EBP-COQ Prof©), manufactured and weighted by Ruzafa-Martinez et al., was used in the present study [7]. The EBP-COQ Prof© scale consists of 35 items that attempt to assess the competence of nursing staff by focusing on the attitudes, skills, and knowledge around Evidence-Based Practice (EBP), as well as the utilization of EBP in clinical practice. These 35 items are in the form of a 5-point Likert scale, with 1 corresponding to totally disagree, 2 to disagree, 3 to neither agree nor disagree, 4 to agree, and 5 to agree completely. The participants completed sociodemographic questions regarding age (years) and biological sex (male/female).

### 2.2. Translation

The EBP-COQ Prof©, manufactured and weighted by the Spanish partners [7], was translated and culturally weighed according to the “Minimal Translation Criteria” [14]. The process included the independent translation of the original Spanish forward translation by two different people. After this phase, the two translations were compared by a third person who was able to decide between any of the different translation versions in order to obtain an agreed translation (1st reconciliation version). The agreed version was then translated into the language of the original questionnaire (backward translation), i.e., Spanish, by a bilingual person (whose mother tongue was Spanish) who was a professional translator but did not know the standard form of the questionnaire. The backward translation version of the questionnaire was sent to the authors for comments and their comments were incorporated by giving a second version of the questionnaire in Greek (2nd reconciliation version).

The version of the questionnaire that resulted from the translation process was completed by 10 clinical nurses, with Greek as their native language, from the target population in order to assess the apparent validity (face validity), confirming that the scale consists of questions that are consistent with the attributes to be measured and does not result in an incomplete response to the questions or to misleading answers [15]. This procedure allows the verification of the comprehensibility of the questionnaire for Greek speakers. Moreover, the questionnaire was evaluated by 3 experts in the field of EBP, who therefore had the cognitive ability not only to evaluate the tool but also to propose methods of improving it (content validity) [16,17]. The items with a coefficient validity ratio (CVR) of >0.70 were preserved in the final version of the instrument. We also calculated the content validity index (CVI) for the instrument as a whole, considering a value of >0.80 to be adequate.

The original English and the Greek questionnaires are available in the (Appendix A).

### 2.3. Participants

The research was conducted in public hospitals in Crete and Athens (Greece). For the collection of the data, the sampling method of convenience was used. Data collection for the questionnaire took place between September 2020 and February 2021. We distributed 550 questionnaires and 414 were answered (75.3% response rate).

### 2.4. Reliability–Validity

Internal consistency was assessed by Cronbach’s alpha. A Cronbach α coefficient of >0.7 indicates sufficient reliability for research purposes and suggests that the items are interdependent and homogeneous in terms of the construct they measure. For clinical applications an α > 0.8 is desirable.

Intra-rater reliability was determined by calculating the intraclass correlation coefficient (ICC) on the initial assessment and the reassessment after a 2-week interval in a random subgroup of the participants. Values below 0.5 indicate poor reliability, between 0.5 and 0.75 indicates moderate reliability, between 0.75 and 0.9 indicates good reliability, and any value above 0.9 shows excellent reliability. The setting of the minimum sample size for the minimum acceptable reliability of 0.8, a significance level of 5%, a power of 99%, and an expected abandonment rate of 10% was estimated at 19 people. So, 20 people were selected randomly and participated in the intra-rater reliability analysis.

Confirmatory factor analysis (CFA) was conducted to determine the model’s fit with a population of 414 clinical nurses. An adequate or a good fit was indicated by a standardized root mean squared residual (SRMR) of less than or equal to 0.08, a coefficient of determination (CD) greater than or equal to 0.90, and a comparative fit index (CFI) greater than or equal to 0.90.

Exploratory factor analysis (EFA) was conducted to identify a viable factor structure. EFA, using the principal component extraction method with varimax rotation, was conducted to determine the factor structure of the 35 items of the EBP-COQ Prof questionnaire (Greek version). A Bartlett’s test of sphericity was conducted to examine the correlation among the items. The Kaiser–Meyer–Olkin (KMO) measure of sampling adequacy was computed to quantify the degree of intercorrelations among the variables and the appropriateness of the factor analysis. To justify the factor analysis, the KMO values should exceed 0.60. For the final model, we used the combination of the following selection criteria: (a) sample size of ≥250, (b) scree plot, (c) each factor contains items with loading of ≥0.50, while at the same time loading of <0.50 for all other factors, (d) each factor contains at least three items with loading of ≥0.50, and (e) the proportion of the total variance explained by the retained factors should be at least 60%.

### 2.5. Data Analysis

We performed the statistical analysis using STATA software (version 12.; Stata Corporation, College Station, TX, USA) for the confirmatory factor analysis and SPSS statistical software (version 25; SPSS, Chicago, IL, USA) for the remainder. Continuous variables were expressed as mean ± standard deviation and categorical variables were expressed as numbers (percentages). To check the condition of normality, the Shapiro–Wilk test and the study of the graphic representations “Normal Q-Q plot”, “Detrended Normal Q-Q plot”, and “Box Plot” were used. The significance level alpha was set at 0.05.

### 2.6. Ethical Considerations

Permission to use the questionnaire was obtained from the original author of the instrument. This research was reviewed and approved by the Hellenic Mediterranean University Ethics Committee with number 28/18.01.21. This survey was carried out in full compliance with the new General Data Protection Regulation (GDPR) [EU 2016/679] 25.5.2018 on sensitive personal data. Prior to its implementation, the relevant licenses were secured by the respective services. The data collected were anonymous; their use was made solely for the purposes of the survey and for access to them by the lead researcher. The participants consented in writing, having been fully informed that the procedure was anonymous, that their personal data and answers would be used exclusively for research purposes, and that at any time they would be able to leave.

## 3. Results

Table 1 presents the demographic characteristics of the respondents.

The sample comprised 414 participants, 354 (85.5 %) females and 60 (14.5 %) males, aged between 22 and 55 years (M = 43, SD = 8.4). The participants were recruited from 10 hospitals of Greece (Crete = 6, Athens = 4). 

The CVR results showed that 100% of the items (n = 35) were acceptable. All the factors were considered reliable, with values of Cronbach’s alpha ranging from 0.918 to 0.952.

The ICC between the initial assessment and the reassessment of the test was 0.997 (CI 95% 0.994–0.999). This coefficient indicates that scores on the Greek EBP-COQ Prof were moderately consistent between the two occasions.

**Figure 1:**
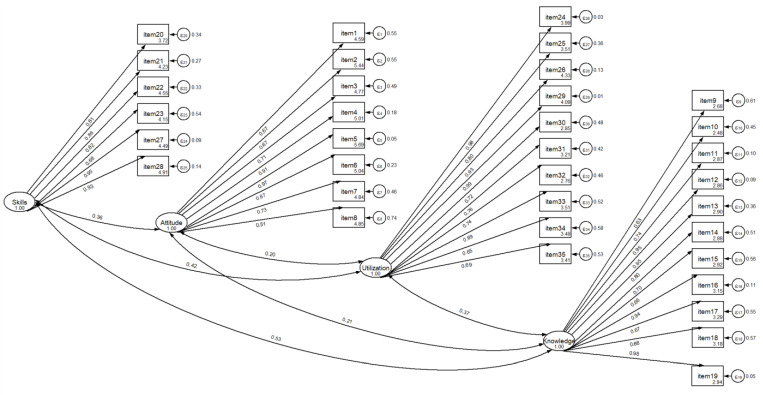
Confirmatory factor analysis of the Greek EBP-COQ Prof.

A four-factor model was conducted by CFA (Figure 1), giving acceptable global fit indices. The resulting global fit indices (SRMR = 0.08, CD = 1.00, and CFI = 0.82) showed that the 35 items in the four-factor solution proposed by the primary researchers should be accepted for the Greek EBP-COQ Prof.

**Figure 2:**
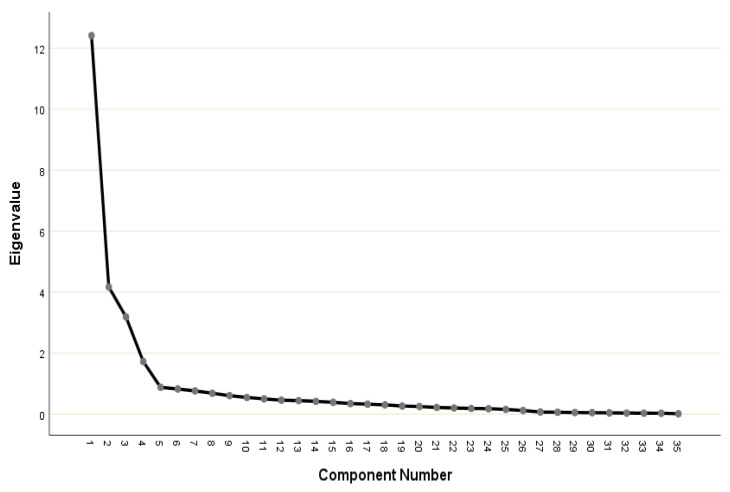
Scree plot.

The Bartlett test of sphericity was 16,483.308 (*p* < 0.001). The Kaiser–Meyer–Olkin measure of sampling adequacy was 0.906, showing that the data were suitable for factor analysis. The 35 items were analyzed via the principal component extraction method, using a varimax rotation. According to the criteria, four factors were identified (Figure 2).

The four-factor solution derived in our study consisted of 35 items (Table 2). The Bartlett’s test of sphericity and the Kaiser–Meyer–Olkin index (KMO) were computed. The Bartlett’s test was significant [χ^2^(595) = 16,483.308, *p* < 0.001], and the KMO index was 0.906; therefore, the data were judged to be appropriate for factor analysis. The four-factor solution had: (1) zero items with salient loadings (≥0.50) on more than one factor; (2) zero items with no salient loading on any factor; and (3) well-defined salient loading per factor (i.e., factor 1 had 11 items and factor 2 had 10 items). With regard to the interpretability of the resulting factor structures and Thurstone’s criteria, the four-factor model was found to be the most suitable for the Greek EBP-COQ Prof. These factors were: (1) knowledge, (2) utilization, (3) attitude, and (4) skills. The total explained variance was 69.6 %. The first factor (items 9–19) accounts for 27.4% of the total variance; the second factor (items 24–26 and 29–35) accounts for 19.1%; the third factor (items 1–8) accounts for 11.5%; and the fourth (items 20–23 and 27–28) accounts for 11.6%

## 4. Discussion

The present study presents the results from the validation of the EBP-COQ Prof Greek version, which took place in a more significant era that examined the association between nurses and EBP [12]. Evidence-based practice is the key research element of the nursing profession and illustrates the basis of all nursing practices and methods. For the purpose of the research, the EBP-COQ Prof Greek version was used, and it was tested in a large sample of nurses working in different public domains. This tool was translated and validated to extract accurate data on the EBP accuracy of nurses. The EBP-COQ Prof Greek version is a psychometric tool composed by Ruzafa-Martinez et al. [7]. The EBP-COQ Prof instrument utilizes the four dimensions that the original authors proposed. These dimensions are attitude, knowledge, skills, and research utilization. The current study explores the validity and sensitivity of this tool in a Greek nursing sample.

The results from the statistical analysis showed that the mean age of the participants was 43 years old. The majority of them were female. The four factors (attitude, knowledge, skills, and research utilization) have a strong association between them due to the factor analysis, which is confirmed by the outcome of previous studies [18,19,20]. Moreover, the Cronbach’s value also shows a high satisfaction between the components, and the final PCA application confirms that EBP is equally composed of the four subscales. The results from previous studies show that EBP utilization depends on education, English fluency, and computer skills [7,21]

There is indeed a gap in global research on EBP, and it is only in the last decades that it has been promoted in nurse education by institutions globally. EBP education affects the nursing practice and nursing culture and has gained the attention of scientists working in the era of evidence-based nursing [22]. As the scientific community states, although nurses are familiar with EBP and believe in its value there is an imperative need for an effective EBP educational approach in the higher education at the undergraduate and postgraduate level [23]. In addition, the promotion and the enhancement of nurses’ EBP lifelong learning is crucial for the implementation of high-quality care in clinical practice [22,24,25]. To ensure that nurses provide high-quality healthcare services, there is an urgent need for nurses to achieve evidence-based nursing practices [21]. Some global strategies to support evidence searching by nurses include interventions such as the creation of a system-wide online EBP education plan; the promotion of free and accessible EBP massive open online courses; and the promotion of best practices online and at international forums [6,22]. Moreover, it is essential and necessary for nurse leaders to share the vision of EBP implementation, using education and by enhancing mentoring to young nurses. More research is needed to structure a domain that disseminates the usage of evidence-based nursing practices.

## 5. Conclusions

The current study revealed that the EBP-COQ Prof GR version is a valid tool which can be used for further research investigation of the registered nurses’ EBP competences. To test the validity of the EBP-COQ Prof instrument in a sample of nurses working in Greek hospitals, four domains were explored by the original EBP-COQ Prof instrument. The study concluded that the EBP competence is affected by the four dimensions of attitude, knowledge, skills, and research utilization. This interprets the dimensions that must be encouraged and synthesized to promote evidence-based practice in nursing and to structure the basis of the nursing profession in the future.

## Figures and Tables

**Table 1:** Respondents’ socio-demographic characteristics.

		n (n%)
**Age (Years)**		43 (8.4) *
**Biological sex**	**Male**	60 (14.5)
	**Female**	354 (85.5)
**Master**	**No**	312 (75.5)
	**Yes**	101 (24.5)
**PhD**	**No**	403 (97.8)
	**Yes**	9 (2.2)
**Years of work**		15.6 (9.3) *

* S mean (standard deviation).

**Table 2:** Rotated Component Matrix.

	Factors			
	1	2	3	4
Item 1			0.718	
Item 2			0.709	
Item 3			0.759	
Item 4			0.869	
Item 5			0.909	
Item 6			0.837	
Item 7			0.776	
Item 8			0.638	
Item 9	0.690			
Item 10	0.795			
Item 11	0.881			
Item 12	0.885			
Item 13	0.805			
Item 14	0.710			
Item 15	0.663			
Item 16	0.928			
Item 17	0.647			
Item 18	0.608			
Item 19	0.920			
Item 20				0.744
Item 21				0.730
Item 22				0.775
Item 23				0.694
Item 24		0.905		
Item 25		0.895		
Item 26		0.875		
Item 27				0.837
Item 28				0.866
Item 29		0.913		
Item 30		0.819		
Item 31		0.820		
Item 32		0.743		
Item 33		0.545		
Item 34		0.646		
Item 35		0.619		

## Data Availability

The data presented in this study are available on request from the corresponding author.

## Appendix A. Questionnaire to Evaluate the Competency in Evidence-Based Practice of Registered Nurses (EBP-COQ Prof©) in Greek and English Language

**Table:**

**Item**	**Question**	**Διαφωνώ απόλυτα** **Strongly Disagree**	**Διαφωνώ** **Disagree**	**Oύτε συμφωνώ** **Oύτε διαφωνώ** **Neither Agree nor Disagree**	**Συμφωνώ** **Agree**	**Συμφωνώ απόλυτα** **Strongly Agree**
**1**	H ΠΒΕ συμβάλλει στη λήψη αποφάσεων στην κλινική πρακτική/EBP helps decision-making in clinical practice	1	2	3	4	5
**2**	Με ευχαριστεί να διαθέτω επιστημονικές ενδείξεις που στηρίζουν τις νοσηλευτικές διεργασίες που εφαρμόζω/I am grateful for the availability of scientific evidence that supports the care I practice	1	2	3	4	5
**3**	H ΠΒΕ αυξάνει την επαγγελματική αυτονομία του νοσηλευτή/τριας/EBP increases the autonomy of the nursing profesión	1	2	3	4	5
**4**	Με ευχαριστεί ή θα με ευχαριστούσε να εφαρμοζόταν η ΠΒΕ στη δομή όπου εργάζομαι/I am grateful or would be grateful for the application of EBP in my work center	1	2	3	4	5
**5**	Aυτή τη στιγμή η ΠΒΕ είναι μια από τις επαγγελματικές μου προτεραιότητες/EBP is one of my professional priorities right now	1	2	3	4	5
**6**	Με την εφαρμογή της ΠΒΕ προάγεται η φροντίδα των ασθενών/The application of EBP improves patient care	1	2	3	4	5
**7**	Είμαι διατεθειμένος/η να κάνω μεγαλύτερη προσπάθεια για να εφαρμόσω την ΠΒΕ στην κλινική πρακτική μου/I am willing to make a greater effort to apply EBP in my clinical practice	1	2	3	4	5
**8**	Πιστεύω ότι θα έπρεπε να εκπαιδευτώ περισσότερο σχετικά με την ΠΒΕ/I believe I should gain more training in EBP	1	2	3	4	5
**9**	Γνωρίζω πώς να διαμορφώνω ερωτήματα σύμφωνα με τον τύπο PICO (πληθυσμός, παρέμβαση, σύγκριση και έκβαση)/I know how to formulate clinical questions structured according to the PICO question (patient, intervention, comparison, and outcome)	1	2	3	4	5
**10**	Γνωρίζω τις βασικές διαδικτυακές σελίδες και βιβλιογραφικές βάσεις με πληροφορίες που έχουν ήδη υποβληθεί σε κριτική αξιολόγηση (όπως PubMed, Scopus, NICE)/I know the main websites with information that has already been critically evaluated (Cochrane, NICE, Guiasalud…)	1	2	3	4	5
**11**	Γνωρίζω τους παράγοντες που καθορίζουν την ποιότητα τις ποσοτικής έρευνας/ I know the aspects that determine the quality of quantitative research	1	2	3	4	5
**12**	Γνωρίζω τους παράγοντες που καθορίζουν την ποιότητα της ποιοτικής έρευνας/I know the aspects that determine the quality of qualitative research	1	2	3	4	5
**13**	Γνωρίζω τα επίπεδα ενδείξεων που αφορούν το κάθε είδος ερευνητικού σχεδιασμού/I know the evidence level of the different designs of research studies	1	2	3	4	5
**14**	Γνωρίζω τους βαθμούς σύστασης (recommendation) που υποστηρίζουν την εφαρμογή των παρεμβάσεων στην υγεία/I know the degrees of recommendation that endorse the introduction of health interventions	1	2	3	4	5
**15**	Γνωρίζω την σημασία των βασικών μέτρων μέτρων συχνότητας και όρους επιδημιολογικούς (επιπολασμός, επίπτωση, RR, OR,κλπ.)/I know the meaning of the main measures of association and effect size (Student’s t, chi-square, RR, OR, and NNT, etc.)	1	2	3	4	5
**16**	Νιώθω ικανός/ή να θέσω ένα κλινικό ερώτημα, ώστε να ξεκινήσω βιβλιογραφική αναζήτηση/I feel able to pose a clinical question to initiate a bibliographic search for scientific evidence	1	2	3	4	5
**17**	Νιώθω ικανός/ή να πραγματοποιώ βιβλιογραφική αναζήτηση με δομημένο τρόπο στις κύριες βάσεις δεδομένων/I feel able to carry out structured bibliographic searches in the main databases	1	2	3	4	5
**18**	Νιώθω ικανός/ή να αξιολογήσω την μεθοδολογική ποιότητα ενός επιστημονικού άρθρου/I feel able to evaluate the methodological quality of a scientific article	1	2	3	4	5
**19**	Νιώθω ικανός/ή να ερμηνεύσω τη σημασία και την ακρίβεια των αποτελεσμάτων ενός επιστημονικού άρθρου/I feel able to interpret the effect size and precision of the results of a scientific article	1	2	3	4	5
**20**	Νιώθω ικανός/ή να εκτιμήσω την δυνατότητα εφαρμογής των αποτελεσμάτων ενός επιστημονικού άρθρου στη δομή όπου εργάζομαι/I feel able to evaluate the applicability of the results of a scientific article in my work center	1	2	3	4	5
**21**	Νιώθω ικανός/ή να αναλύσω ένα κλινικό πρόβλημα από την αξιολόγηση του ασθενούς και/ή την αξιολόγηση της έκβασης της υγείας τους/I feel able to analyze a clinical problem based on the assessment of the patient and/or the evaluation of his/her health outcomes	1	2	3	4	5
**22**	Νιώθω ικανός/ή να μεταφέρω στους συναδέλφους μου τα αποτελέσματα που αποκτώ κατά την κλινική πρακτική μου/I feel able to communicate to my colleagues the results obtained with my clinical practice	1	2	3	4	5
**23**	Νιώθω ικανός/ή να συνεργαστώ (ή να ηγηθώ) για αλλαγές στην κλινική πρακτική στη δομή όπου εργάζομαι/I feel able to collaborate in (or lead) changes in clinical practice in my work center	1	2	3	4	5
**24**	Στο τμήμα όπου εργάζομαι πραγματοποιούνται παρεμβάσεις βασισμένες σε επιστημονικές ενδείξεις/Interventions based on scientific evidence are performed in my work center	1	2	3	4	5
**25**	H πλειοψηφία των παρεμβάσεων που βασίζονται σε ενδείξεις και πραγματοποιούνται στην εργασία μου προτείνονται από θεσμούς όπως το Υπουργείο Υγείας, ΕOΔΥ κτλ./The majority of evidence-based interventions in my work center are proposed by my health organization	1	2	3	4	5
**26**	H πλειοψηφία των παρεμβάσεων που βασίζονται σε ενδείξεις που πραγματοποιούνται στην εργασία μου προτείνονται από τους νοσηλευτές/τριες του τμήματος/The majority of evidence-based interventions in my work center are proposed by nurses in the unit.	1	2	3	4	5
**27**	Λαμβάνω υπόψη τις προτιμήσεις των ασθενών ή και των συγγενών στην κλινική μου πρακτική/I take account of the preferences of patients and/or family members in my clinical practice	1	2	3	4	5
**28**	Στην λήψη κλινικών αποφάσεων λαμβάνω υπόψη την επαγγελματική μου εμπειρία/I take account of my professional experience in clinical decision-making	1	2	3	4	5
**29**	Για την κλινική μου πρακτική συμβουλεύομαι επιστημονικές ενδείξεις (κατευθυντήριες οδηγίες, συστηματικές ανασκοπήσεις, πρωτότυπες έρευνες κλπ.)/I consult scientific evidence (clinical practice guidelines, systematic reviews, original studies, etc.) for my clinical practice	1	2	3	4	5
**30**	H δομή στην οποία εργάζομαι κοινοποιεί συχνά στους νοσηλευτές/τριες και διαχέει πληροφορία σχετικά με τα αποτελέσματα που επιτεύχθηκαν από το τμήμα/My institution regularly supplies the nurses with the results obtained by the unit	1	2	3	4	5
**31**	Aναλύω με τους συναδέλφους μου τα αποτελέσματα που έχουν επιτευχθεί μετά την αξιολόγηση των νοσηλευτικών παρεμβάσεων/I analyze with my colleagues the results obtained after evaluation of our care	1	2	3	4	5
**32**	Χρησιμοποιώ έγκυρα εργαλεία (ερωτηματολόγια, τεστ, δείκτες) για να αξιολογήσω τα αποτελέσματα της κλινικής πρακτικής μου/I use validated instruments (questionnaires, tests, indexes, etc.) to evaluate the results of my clinical practice	1	2	3	4	5
**33**	Διατηρώ επικαιροποιημένη την κλινική μου πρακτική σχετικά με πληροφορίες που προέρχονται από κατευθυντήριες οδηγίες, συστηματικές ανασκοπήσεις και άλλες ενδείξεις/I keep my clinical practice updated with information from clinical practice guidelines, systematic reviews, and other evidence	1	2	3	4	5
**34**	Στη δομή όπου εργάζομαι λαμβάνονται αποφάσεις που στηρίζονται σε επιστημονικές ενδείξεις και όχι τόσο στην συνήθεια/παράδοση/εθιμοτυπία/In my work center, the decisions taken are based on scientific evidence rather than custom	1	2	3	4	5
**35**	Στη δομή όπου εργάζομαι συνεργάζομαι, προκειμένου η ΠΒΕ να αποτελέσει μέρος της κουλτούρας του οργανισμού/In my work center, I collaborate in making EBP part of the culture of my organization	1	2	3	4	5
